# Exploring the therapeutic potential of *Tetrastigma bracteolatum* (Wall.) Planch. methanol extract and its different fractions: *in vitro, in vivo,* and *in silico* approaches

**DOI:** 10.3389/fphar.2026.1745518

**Published:** 2026-03-24

**Authors:** Fatima Anjum Faruquee, Tufael Ahmed, Baudrul Mohammad Shahjalal, Utsab Acharya, Milan Rai, Md. Kauser Miah, Mohammad A. Rashid, Safaet Alam, Yuan Seng Wu, Raquibul Hasan, Md. Moklesur Rahman Sarker, Mohd Fahami Nur Azlina, Mohsin Kazi

**Affiliations:** 1 Department of Pharmacy, State University of Bangladesh, Dhaka, Bangladesh; 2 Department of Biomedical Engineering, University of Mississippi, Oxford, MS, United States; 3 Department of Computer and Information Science, Gannon University, Erie, PA, United States; 4 Phytochemical Research Laboratory, Department of Pharmaceutical Chemistry, University of Dhaka, Dhaka, Bangladesh; 5 Chemical Research Division, BCSIR Dhaka Laboratories, Bangladesh Council of Scientific and Industrial Research (BCSIR), Dhaka, Bangladesh; 6 Department of Biomedical Sciences, Sir Jeffrey Cheah Sunway Medical School, Faculty of Medical and Life Sciences, Sunway University, Sunway City, Malaysia; 7 Sunway Microbiome Centre, Faculty of Medical and Life Sciences, Sunway University, Sunway City, Malaysia; 8 Department of Pharmaceutical Sciences, College of Pharmacy, Mercer University, Atlanta, GA, United States; 9 Pharmacology and Toxicology Research Division, Health Med Science Research Network, Dhaka, Bangladesh; 10 Pharmacology Department, Faculty of Medicine, Universiti Kebangsaan Malaysia (The National University of Malaysia), Bangi, Selangor, Malaysia; 11 Department of Pharmaceutics, College of Pharmacy, King Saud University, Riyadh, Saudi Arabia

**Keywords:** analgesic, antidiarrheal, antihyperglycemic, anti-oxidant, membrane stabilizing, *Tetrastigma bracteolatum*, thrombolytic

## Abstract

**Introduction:**

Tetrastigma bracteolatum: (Wall.) Planch, an indigenous plant of Bangladesh, is traditionally used for pain management. This study aimed to determine its biological activities through an integrated *in-vitro*, *in-vivo*, and *in silico* approaches.

**Methods:**

Crude methanol extract was prepared using the maceration technique and fractionated into different solvent fractions. Antioxidant, thrombolytic, and membrane-stabilizing activities were assessed *in-vitro*, while antidiabetic, antidiarrheal, CNS-stimulating, and analgesic activities were performed *in-vivo* using Swiss albino mice.

**Results:**

Among the fractions, the chloroform-soluble fraction (CSF) exhibited the highest phenolic content and membrane stabilizing activity, while the aqueous-soluble fraction (AQSF) demonstrated strong antioxidant properties. The petroleum ether fraction showed significant cytotoxic activity. The crude methanol extract exhibited potent antidiarrheal and hypoglycemic effects in mice, with efficacy comparable to standard drugs. The extract significantly exhibited CNS stimulating activity as well. Additionally, it demonstrated analgesic activity by significantly prolonging pain response times and reducing chemically induced pain behaviors in mice. *In-silico* docking studies revealed strong binding affinities of the extract’s compounds against key biological targets involved in inflammation, pain modulation, and metabolic regulation. Moreover, toxicity predictions indicated that all compounds were non-toxic and free from carcinogenic effects.

**Discussion:**

These findings suggest that *T. bracteolatum* possesses promising antioxidant, membrane-stabilizing, antidiarrheal, antihyperglycemic, antidepressant, and analgesic properties, supporting its potential therapeutic applications.

## Introduction

Despite significant advancements in modern medicine over the 20th century, it is estimated that nearly one-third of the global population still lacks access to affordable medications. This disparity has contributed to the growing popularity of traditional, complementary, and alternative medicine (TCAM) in both high-income and low-to middle-income countries. In Bangladesh, traditional medicine continues to play a central role in primary healthcare delivery (Ahmed and Azam, 2014). Traditional healers, locally known as *Kabiraj*, provide services across both urban and rural settings, with a stronger presence and reliance observed in rural communities. These practitioners primarily depend on medicinal plants for therapeutic purposes (Hossan et al., 2010).

Oxidative stress arises from an imbalance between the generation of reactive oxygen species (ROS) and the capacity of biological antioxidant defense systems, leading to oxidative damage of lipids, proteins, and nucleic acids in cells and tissues. This process is widely recognized as a fundamental mechanism underlying the development and progression of numerous chronic and degenerative disorders, including cardiovascular diseases, cancer, neurodegenerative conditions, metabolic syndromes, and inflammatory disorders. Given the limitations and potential adverse effects associated with long-term use of synthetic antioxidants, increasing attention has been directed toward natural antioxidants as safer and biologically compatible alternatives for maintaining redox homeostasis and protecting tissue integrity.

Natural products derived from fungi and plants represent an important source of antioxidant compounds with diverse biological activities. Edible and medicinal phyto sources have been reported to possess substantial antioxidant capacity, reflected by reduced oxidative stress indices, enhanced radical scavenging activity, and protection against DNA damage, supporting their potential role in health promotion and disease prevention (Bal et al., 2019; Sevindik et al., 2017; Selamoglu et al., 2020). These effects are largely attributed to their rich phenolic content and other bioactive constituents capable of modulating oxidative pathways in biological systems.

In addition to dietary sources, specific natural bioactive compounds and products have shown tissue-targeted antioxidant effects. Natural anthraquinones such as physcion and physcion-8-O-β-D-glucopyranoside exhibit antioxidant-mediated anticancer activities by regulating oxidative stress–related signaling pathways and cellular apoptosis (Adnan et al., 2021). Collectively, these findings emphasize the growing importance of natural antioxidants in mitigating oxidative stress and preserving tissue health, providing a strong scientific rationale for further investigation into their biological properties and therapeutic potential.

Traditional medicine including herbal preparations, functional foods, and nutraceuticals has gained recognition as a promising alternative or complement to conventional pharmacotherapy. A large body of scientific evidence, derived from *in vitro*, *in vivo*, and clinical studies, supports the bioactivity of various natural compounds in managing chronic and infectious diseases. These include antidiabetic (Chen et al., 2018; Chen et al., 2019; Ullah et al., 2017; Khan et al., 2019; Kifayatullah et al., 2017a; Kifayatullah et al., 2017b; Sarker et al., 2015; Shah et al., 2016; Rouhi et al., 2017), anticancer [12–14] (Shajib et al., 2018; Sheikh et al., 2017a; Sheikh et al., 2017b), immunomodulatory (Goto et al., 2010; Sarker et al., 2012a; Sarker, 2012; Sarker et al., 2014; Sarker et al., 2012b; Sarker et al., 2011; Sarker et al., 2012c; Sarker et al., 2016; Sarker and Gohda, 2013), antihyperlipidemic and anti-obesity (Kazemipoor et al., 2015; Sarker et al., 2015), analgesic (Nesa et al., 2018), anti-inflammatory (Imam et al., 2013; Kamarudin et al., 2017), antioxidant (Karim et al., 2015), neuroprotective (Das et al., 2017), and antimicrobial (Yasmin et al., 2009) effects, among others.

One such medicinal plant is *Tetrastigma bracteolatum* (Wall.) Planch., a member of the Vitaceae family, which has been traditionally used for pain relief, particularly in the hilly areas of Bangladesh such as Bandarban. This species is a robust woody climber characterized by slender branches and glabrous leaves and stems (Borah, 2014). *Tetrastigma*, a genus within the grape family, is locally referred to as “Golgotilata,” while the Chakma community calls it “Khurangulludi” (The Plant list, 2010). The plant is predominantly found in the Chittagong Hill Tracts, Cox’s Bazar, Sylhet, and Moulvibazar districts (Hossan et al., 2010). Among its traditional applications, the Chakma people frequently apply a paste of the leaves to the forehead to relieve headaches (Borah, 2014; Khisha et al., 2012), whereas the Chiru tribe of Manipur, India, uses decoctions of the leaves and fruits to treat digestive disorders (Rajkumari et al., 2013). Despite its ethnomedicinal significance, there is currently a lack of published scientific data validating the therapeutic efficacy, optimal dosing, or potential toxicity of *T. bracteolatum*. Therefore, systematic pharmacological and toxicological evaluations are essential to substantiate its traditional uses and to assess its potential as a source of novel therapeutic agents.

This study aims to investigate the medicinal potential of *T. bracteolatum* (Wall.) Planch. through a combination of *in vitro*, *in vivo*, and *in silico* approaches. Methanolic extracts and various organic fractions of the plant were subjected to *in vitro* assays to assess thrombolytic, antioxidant, membrane-stabilizing, and cytotoxic activities. Additionally, *in vivo* experiments using Swiss albino mice (6–8 weeks old, both sexes) were conducted to evaluate analgesic, antihyperglycemic, and antidiarrheal properties. In silico molecular docking studies were also performed to explore the interactions between the plant’s bioactive constituents and their putative biological targets.

## Materials and methods

### Collection and identification of the plant

Fresh specimens of *Tetrastigma bracteolatum* (Wall.) Planch. were collected in May 2018 from the hilly regions of Chittagong Division, Bangladesh, by Fatima Anjum Farooque. Botanical identification was confirmed by Dr. Sordar Nasiruddin and Dr. Mahbuba Sultana at the Bangladesh National Herbarium (Mirpur, Dhaka), where a voucher specimen has been archived under accession number 46512.

### Preparation and fractionation of extracts

After cleaning, the whole plant material was shade-dried and coarsely powdered. A total of 400 g of the powdered sample was macerated in 1,600 mL of methanol and stored in an amber glass container for 7 days at room temperature with periodic agitation. Following extraction, the solution was filtered first through cotton and then through Whatman No. 1 filter paper. The filtrate was concentrated using a rotary evaporator at 40 °C to obtain a semi-solid methanolic extract (METB). A portion of the crude extract (5 g) was further partitioned using a stepwise polarity gradient, based on a modified Kupchan method. The sample was dissolved in 10% aqueous methanol and subsequently fractionated with petroleum ether, dichloromethane, chloroform, and distilled water to yield the petroleum ether-soluble fraction (PESF), dichloromethane-soluble fraction (DCMSF), chloroform-soluble fraction (CSF), and aqueous-soluble fraction (AQSF).

### Chemicals and reagents

Glibenclamide and loperamide were generously provided by Square Pharmaceuticals Ltd. (Dhaka, Bangladesh), while diclofenac sodium and normal saline were supplied by Incepta Pharmaceuticals Ltd. (Dhaka, Bangladesh). Vincristine sulfate and morphine sulfate were purchased from Sigma-Aldrich (Germany). DPPH (2,2-diphenyl-1-picrylhydrazyl) and BHT (butylated hydroxytoluene) were procured from Merck (India). All other chemicals used were of analytical grade and purchased locally.

### Experimental animals

Swiss albino mice (both sexes, 5–6 weeks age, 25–35 g) were sourced from the Animal Breeding and Pharmacology Laboratory, Jahangirnagar University, Bangladesh. Animals were housed under standard laboratory conditions (22–24 °C, 12 h light/dark cycle) with food and water access *ad libitum*. Mice were allowed a 1-week acclimatization period prior to experiments. All experimental protocols were approved by the Animal Ethics Committee of the State University of Bangladesh (Approval No. 2019-06-10/SUB/A-ERC/0005). Ethical guidelines from FELASA, the Basel Declaration, and ICLAS were strictly followed. The 3Rs (Replacement, Reduction, and Refinement) were applied throughout the study. At the end of the experiments, euthanasia was performed by administering an intraperitoneal overdose of ketamine HCl (100 mg/kg) and xylazine (10 mg/kg) (Davis, 2001).

### Determination of total phenolic contents

The total phenolic content was determined using the Folin–Ciocalteu colorimetric method (Ainsworth and Gillespie, 2007). Briefly, the diluted Folin–Ciocalteu reagent (2.5 mL) was mixed with 2 mL of the extract, followed by the addition of sodium carbonate (7.5%, 2 mg/mL). After incubation at 45 °C for 15 min, absorbance was measured at 765 nm using a UV–Vis spectrophotometer. Results were expressed as mg gallic acid equivalents (GAE) per g of extract.

### Determination of antioxidant activity by the free radical scavenging DPPH method

The antioxidant activity was measured using the DPPH radical scavenging method described by Brand-Williams et al. (Brand-Williams et al., 1995). A 100 μL of various concentrations of the extract (0.977–500 μg/mL) were mixed with 3 mL of DPPH solution (20 μg/mL). The mixtures were kept in the dark for 30 min at room temperature, and absorbance was read at 517 nm. The percentage of inhibition was calculated using the formula:

$$\%\text{ inhibition}=\left[\left(A_{0}-A_{1}\right)/A_{0}\right]\times 100$$

where A_0_ is the absorbance of the control and A_1_ is the absorbance of the sample. The IC_50_ value was derived from the plotted inhibition curve.

### Determination of cytotoxic activity by brine shrimp lethality bioassay

The cytotoxic effect of METB and its fractions was evaluated using the brine shrimp lethality bioassay (Meyer et al., 1982). Stock solutions of the test samples were prepared in DMSO and serially diluted to obtain final concentrations ranging from 0.781 to 400 μg/mL. Each solution (50 µL) was added to test tubes containing 5 mL of simulated seawater and 10 brine shrimp nauplii. After 24 h of incubation at room temperature, the number of surviving nauplii was counted. LC_50_ values were determined using a regression analysis of log concentration versus percent mortality. Vincristine sulfate served as the reference drug.

### Determination of thrombolytic activity

Thrombolytic activity was assessed following clot lysis, using the method described by Prasad et al. (Prasad et al., 2007). Venous blood (5 mL) from healthy volunteers was allowed to clot in pre-weighed microcentrifuge tubes at 37 °C for 45 min. After removal of serum, each clot was treated with 100 µL of test extract (20 mg/mL), streptokinase (30,000 IU/100 μL, positive control), or distilled water (negative control). After incubation for 90 min at 37 °C, released fluid was removed and tube weights were recorded.

$$\%\text{ Clot lysis}=\left[\left(\text{Weight of released clot}\right)/\left(\text{Initial clot weight}\right)\right]\times 100$$

### Determination of membrane stabilizing activity in red blood cells

The protective effect of the extracts against RBC hemolysis was evaluated under hypotonic and thermal stress conditions (Yadav et al., 2024). In the hypotonic solution-induced hemolysis test, erythrocytes were incubated with extract solutions (2 mg/mL) in hypotonic buffer. The mixture was centrifuged at 3,000×g, and absorbance of the supernatant was measured at 540 nm.

$$\%\text{ Hemolysis}=100\times \left[\left({\text{OD}}_{1}-{\text{OD}}_{2}\right)/{\text{OD}}_{1}\right]$$

where OD_1_ is the optical density of the control and OD_2_ is that of the test sample.

In the heat-induced hemolysis assay, samples were incubated at 54 °C and 0–5 °C, then centrifuged and analyzed at 540 nm. Hemolysis was calculated as:

$$\%\text{ Hemolysis}=100\times \left[1-\left({\text{OD}}_{2}\;‐\;{\text{OD}}_{1}\right)/\left({\text{OD}}_{3}\;‐\;{\text{OD}}_{1}\right)\right]$$

where OD_1_, unheated sample, OD_2_, heated sample, OD_3_, negative control.

### Research design for *in vivo* experiments

The experimental Swiss albino male mice were randomly divided into follwing 4 groups (5 mice per group) namely group-1 (negative/disease control group), group-2 (standard drug or positive control group), group-3 (extract dose-1/METB 200 mg/kg), and group-4 (extract dose-2/METB 400 mg/kg).

### Determination of central and peripheral analgesic activity

Central analgesic activity was assessed via the tail flick method (Nesa et al., 2018; Eghianruwa et al., 2020), and peripheral analgesia was evaluated using the acetic acid-induced writhing test (Nesa et al., 2018; Eghianruwa et al., 2020). Swiss albino male mice (6–8 weeks age) were orally treated with METB (200 and 400 mg/kg), morphine sulfate (2 mg/kg, s. c.), or diclofenac sodium (50 mg/kg, oral). For the tail flick test, latency to tail withdrawal was recorded at 30, 60, and 90 min.

$$\%\text{ Pain inhibition}=\left[\left(T_{1}-T_{0}\right)/T_{0}\right]\times 100$$

where T_1_, post-treatment latency and T_0_ = baseline latency.

In the writhing test, acetic acid (1%, 10 mL/kg, i. p.) was administered 40 min after extract administration. The number of writhes in 10 min was counted.

$$\%\text{ Inhibition}=\left[\left(\text{Control}-\text{Test}\right)/\text{Control}\right]\times 100$$

### Anti-diarrheal activity

Antidiarrheal effects were tested using castor oil-induced diarrhea (Kassaw et al., 2025). Swiss albino mice (6–8 weeks age), fasted for 14 h, were orally administered with METB (200 or 400 mg/kg), loperamide (2 mg/kg), or saline. One hour post-treatment, castor oil (1 mL) was administered. Fecal output was monitored for 4 h.

$$\%\text{ Inhibition}=\left[\left(\text{Control}-\text{Test}\right)/\text{Control}\right]\times 100$$

### Oral glucose tolerance test to measure the antihyperglycemic activity of METB

The antihyperglycemic potential of METB was examined in glucose-loaded mice. Swiss albino mice of both sexes (6–8 weeks age) received METB (200/400 mg/kg), glibenclamide (5 mg/kg), or water by oral gavaging. After 20 min, glucose (2 g/kg) was orally administered. Blood glucose was measured at 0, 30, 60, 120, and 180 min using a glucometer (Accu-Chek, Roche) (Nesa et al., 2018; Hasan et al., 2018).

### CNS stimulant activity: Thiopental sodium-induced sleeping time bioassay

The sedative effect was evaluated using the thiopental sodium-induced sleep model (Hassan et al., 2024). Swiss albino mice (6–8 W) received METB (200 or 400 mg/kg) or vehicle through oral route. After 30 min, thiopental sodium (25 mg/kg, i. p.) was administered. Sleep onset and duration were recorded for each animal.

### Statistical analysis

Data are presented as mean ± SEM (standard error means) (n = 3 or 5). One-way ANOVA followed by Dunnett’s Post-Hoc test was performed using SPSS v25. A *P*-value <0.05 was considered statistically significant.

### In-silico protein ligand preparation and molecular docking

**FIGURE 1:**
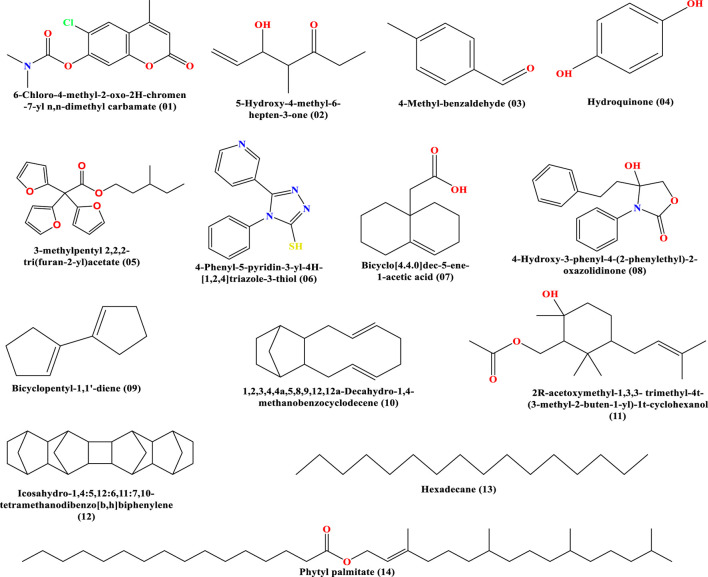
2D chemical structures of the reported molecules.

**FIGURE 2:**
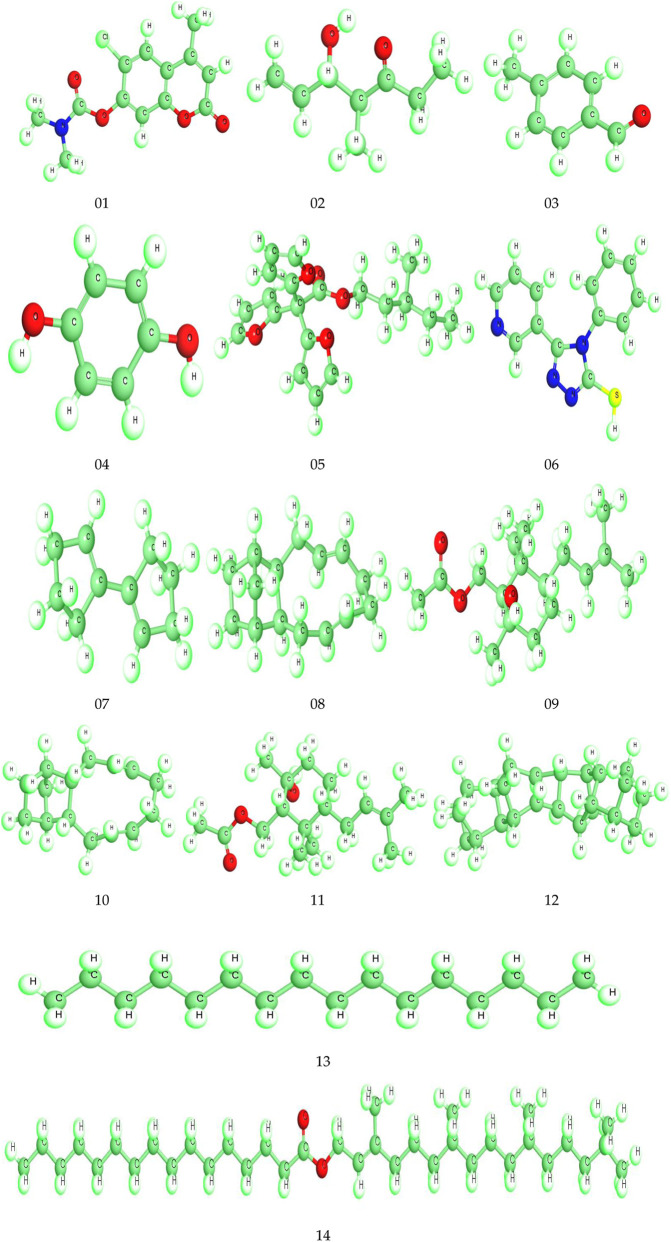
3D optimized structures of the reported molecules.

**FIGURE 3:**
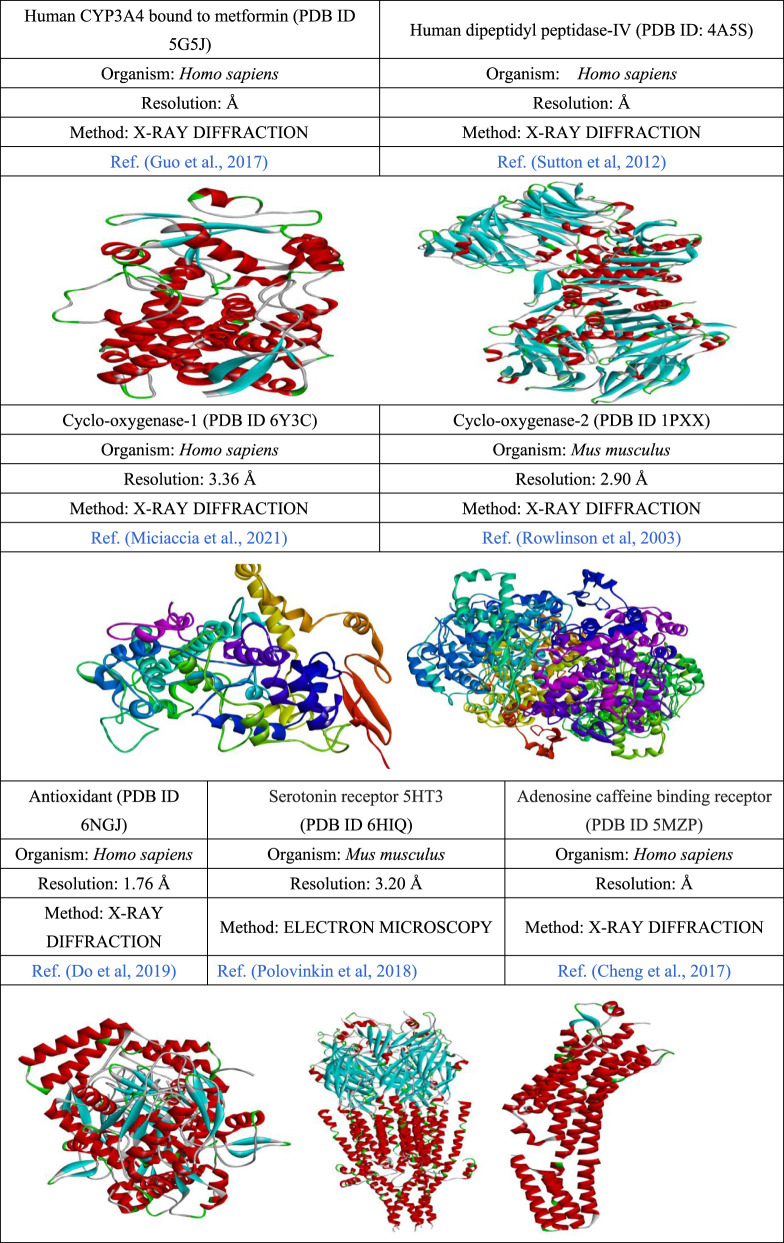
Targeted protein information and tertiary structures.

Chemical structures of selected bioactive compounds were drawn using ChemBioDraw and converted to 3D via Materials Studio 08. Structures were optimized using DFT and saved in SDF format (Kumer et al., 2022a). The 2D and 3D structures of all ligands are shown in Figures 1, 2. The responsible receptor or targeted protein of human CYP3A4 bound to metformin (PDB ID 5G5J), human dipeptidyl peptidase-IV (PDB ID: 4A5S), cyclo-oxygenase-1 (PDB ID 6Y3C), cyclo-oxygenase-2 (PDB ID 1PXX), antioxidant (PDB ID 6NGJ), serotonin receptor 5HT3 (PDB ID 6HIQ), and adenosine caffeine binding receptor (PDB ID 5MZP) were retrieved from the Protein Data Bank as followed by other researchers (Cheng et al., 2017; Do et al., 2019; Guo et al., 2017; Miciaccia et al., 2021; Polovinkin et al., 2018; Rowlinson et al., 2003; Sutton et al., 2012). After that, they were cleaned by Biovia Discovery Studio 2021 to obtain fresh protein (Kumer et al., 2022b). When, the protein is cleaned up completely, these all are exported in PDB format, as shown in Figure 3. Finally, molecular docking was conducted using PyRx AutoDock Vina to estimate binding affinities (Dallakyan and Olson, 2015). In a previous study, a total of 14 phytocomounds were identified in the ethanol extract of *Tetrastigma bracteolatum* (Wall.) identified through GC–MS analysis (Islam et al., 2023). We have conducted molecular docking of those compouds to find out the possible molecular mechanisms in support of the therapeutic properties of the extracts.

### ADME/T, Lipinski’s role and drug-likeness analysis

Drug discovery is a complex process, and most drug candidates cannot reach the final stages due to lower potency and unwanted side effects. Therefore, before going to laboratory experiments, *in silico* ADMET analysis may be a useful tool to separate good and bad drug molecules, which may reduce time cost and resources (Daina et al., 2017; Banerjee et al., 2024). Therefore, the ADMET and pharmacokinetic profiles of compounds have been predicted by using an online platform known as the pkCSM web tool (Azzam, 2023). This online tool is considered one of the most trusted websites to predict *in silico* ADMET analysis.

ADMET properties and drug-likeness of the compounds were predicted using the pkCSM online platform (Daina et al., 2017; Banerjee et al., 2024; Azzam, 2023), based on Lipinski’s rule of five to assess oral bioavailability and pharmacokinetic profiles.

## Results

### Total phenolic contents in crude METB and in its different fractions

The total phenolic content varied found from our experiment is ranging from lowest as 0.38 mg of GAE/gm of PESF to as high as 86.70 mg GAE/gm of CSF. The highest phenolic content was found in CSF (86.70 ± 0.29), followed by DCMSF (40.07 ± 0.86), MESF (33.01 ± 0.32), and AQSF (20.52 ± 0.65) mg of GAE/gm of extractives (Table 1).

**TABLE 1:** Total phenolic content, anti-oxidant and cytotoxic activity of various soluble fractions of the crude extract of *Tetrastigma bracteolatum*.

Samples/Standards	Total phenolic content (mg of GAE/g of dried extract)	Antioxidant activity IC_50_ (µg/mL)	Cytotoxic activity LC_50_ (µg/mL)
MESF	33.01 ± 0.32	170.13 ± 2.40	31.53 ± 0.26
PESF	0.38 ± 0.23	80.38 ± 0.43	1.41 ± 0.01
CSF	86.70 ± 0.29	27.74 ± 0.55	12.41 ± 0.16
DCMSF	40.07 ± 0.86	74.49 ± 2.79	51.47 ± 0.29
AQSF	20.52 ± 0.65	7.48 ± 0.05	39.34 ± 0.05
VS (std.)	-	-	0.45 ± 0.00
BHT (std.)	-	21.68 ± 0.41	-

MESF, methanol extract soluble fraction; PESF, pet ether soluble fraction; CSF, chloroform soluble fraction; DCMSF, dichloromethane soluble fraction; AQSF, aqueous soluble fraction; VS, vincristine sulfate; BHT, butylated hydroxytoluene; GAE, gallic acid equivalent.

### Antioxidant activity of METB and its different fractions

The antioxidant activity of METB and its different fractions was determined by measuring its ability to scavenge DPPH free radicals. Our investigation resulted in the highest to lowest free radical scavenging ability of AQSF, CSF, DCMSF, PESF, and MESF, showing IC_50_ values of 7.48 ± 0.05 μg/mL, 27.74 ± 0.55 μg/mL, 74.49 ± 2.79 μg/mL, 80.38 ± 0.43 μg/mL, and 170.13 ± 2.40 μg/mL, respectively. The IC_50_ value of AQSF (aqueous soluble fraction of extract) (7.48 ± 0.05 μg/mL) was found to be stronger than that of BHT (a standard antioxidant used in this experiment) (21.68 ± 0.41 μg/mL). In addition, the antioxidant capacity of CSF (IC_50:_ 27.74 ± 0.55 μg/mL) was comparable to that of BHT (IC_50_: 21.68 ± 0.41 μg/mL) (Table 1).

### Effect of METB and its different fractions on cytotoxic activity on brine shrimp nauplii

The cytotoxic effects of METB and its different fractions on lethality in brine shrimp are presented in Table 1. Among the tested extracts and fractions, the highest cytotoxic activity was exhibited by PESF, followed by CSF, MESF, AQSF, and DCMSF, with LC_50_ values of 1.41 ± 0.01 μg/mL, 12.41 ± 0.16 μg/mL, 31.59 ± 0.26 μg/mL, 39.34 ± 0.05 μg/mL, and 51.47 ± 0.29 μg/mL, respectively. The LC_50_ value of the petroleum ether soluble fraction (PESF) of 1.41 ± 0.01 μg/mL was comparable to that of vincristine sulfate (VS: an anticancer drug used as a standard in this exp.) (0.45 ± 0.00 μg/mL).

### Effect of METB and its different fractions on membrane stabilization of RBCs

**FIGURE 4:**
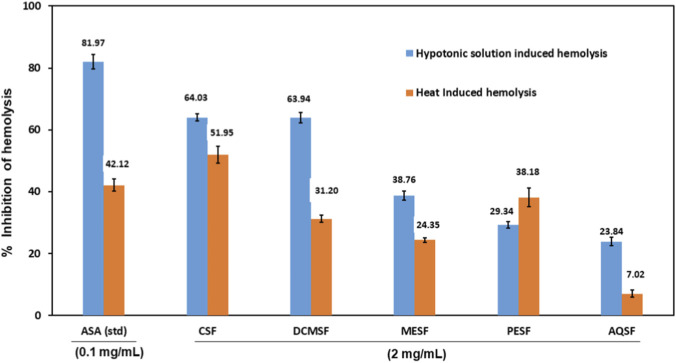
Membrane stabilizing activity of the different extractives of *Tetrastigma bracteolatum* and acetyl salicylic acid as the standard on hypotonic solution-induced hemolysis of the membrane of erythrocytes. ASA, Acetyl salicylic acid (standard); CSF, Chloroform soluble fraction; DCMSF, Dichloromethane soluble fraction; MESF, Methanol extract soluble fraction; PESF, Petroleum ether soluble fraction; AQSF, Aqueous soluble fraction.

The membrane stabilizing activities of METB and its different fractions were determined by hypotonic-solution-induced as well as heat-induced hemolysis. In the case of hypotonic solution-induced hemolysis, the highest membrane stabilizing activity was shown by CSF (64.03% ± 1.16%) inhibition of the RBC membrane compared to the control), followed by DCMSF (63.94% ± 1.77%), MESF (38.76 ± 1.49), PESF (29.34 ± 1.06), and AQSF (23.84 ± 1.35), whereas the standard membrane stabilizing agent aspirin showed 81.97% ± 2.37% inhibition of hemolysis (Figure 4).

Similarly, in the case of heat-induced hemolysis experiments, the highest membrane inhibition was exhibited by CSF (51.95% ± 2.82%), followed by PESF (38.18% ± 3.11%), DCMSF (31.20% ± 1.21%), MESF (24.35% ± 0.77%), and AQSF (7.02% ± 1.11%). Our investigation resulted in the higher membrane stabilizing activity of the chloroform soluble fraction of METB (51.95% ± 2.82%) than that of the standard membrane stabilizing drug aspirin (42.12% ± 2.04%) (Figure 4).

### Thrombolytic activity of METB and its different fractions

**FIGURE 5:**
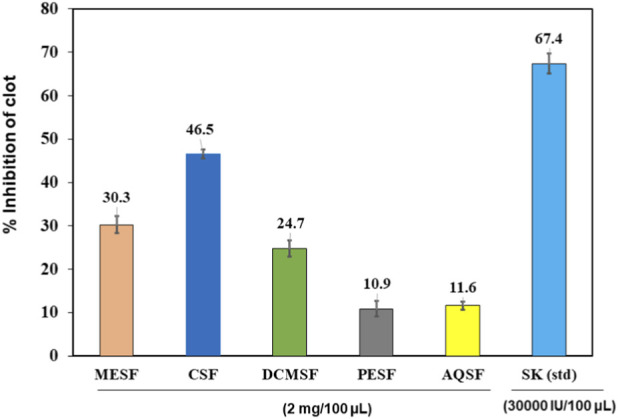
Thrombolytic activity of the different extractives of *Tetrastigma bracteolatum* and streptokinase as the standard. MESF, Methanol extract soluble fraction; CSF, Chloroform soluble fraction; DCMSF, Dichloromethane soluble fraction; PESF, Petroleum ether soluble fraction; AQSF, Aqueous soluble fraction; SK, Streptokinase (standard).

All the extractives showed inhibition of blood clotting in the thrombolytic bioassay, but in all cases, the inhibition ability was lower than that of the standard thrombolytic drug streptokinase (67.36% ± 2.31%). The highest inhibition was exhibited by the CSF (46.46± 1.02%), followed by the MESF (33.3± 2.03%) and DCMSF (24.7± 1.87%) (Figure 5).

### Antidiarrheal activity

The methanol extract of *T. bracteolatum* showed dose-dependent castor oil-induced diarrhea in Swiss albino mice. The methanol extract at doses of 200 and 400 mg/kg reduced diarrhea-related stool defecation by 80.78% (P < 0.001) and 87.18% (P < 0.001), respectively (Table 2).

**TABLE 2:** Effects of *Tetrastigma bracteolatum* crude methanol extract on castor oil-induced diarrhea in mice.

Group	n	Number of stools after 4 h (mean ± SEM)	Inhibition of defecation (%)
Control (normal saline)	5	26.0 ± 0.58	-
Loperamide (std.) (3 mg/kg)	5	4.67 ± 0.67***	82.05%
MESF (200 mg/kg)	5	5.0 ± 1.53***	80.78%
MESF (400 mg/kg)	5	3.33 ± 0.88***	87.18%

Here, *P < 0.05, **P < 0.01 and ***P < 0.001 when compared with the control group. SEM, standard error of mean, MESF, methanol soluble fraction, n, Sample size.

### Evaluation of METB for oral glucose tolerability

The oral glucose tolerance ability of METB was assessed in Swiss albino mice orally administered glucose (2 mg/kg). The glucose levels in mice were determined 30, 60, 120 and 180 min after the administration of oral glucose. METB extract at a dose of 200 mg/kg significantly controlled the boosting of postprandial plasma glucose levels compared to the control, keeping the glucose levels at 6.40 ± 1.39 vs. 14.57 ± 5.14 (P < 0.05) and 5.40 ± 0.56 vs. 15.73 ± 0.03 (P < 0.001) after 60 and 120 min, respectively. Similarly, the plasma blood glucose levels were found to be significantly controlled by METB 400 mg/kg, keeping the glucose levels at 5.67 ± 0.93 vs. 14.57 ± 5.14 (P < 0.05) and 5.87 ± 0.23 vs. 15.73 ± 0.03 (P < 0.001) after 60 and 120 min, respectively. The METB at doses of 200 and 400 mg/kg reduced the boosting of plasma blood glucose levels 2 h after the administration of oral glucose by 65.67% and 62.68%, respectively, compared to the untreated group, whereas the standard antidiabetic drug glibenclamide at a dose of 5 mg/kg reduced plasma glucose levels by 68.02% (Table 3).

**TABLE 3:** Effects of *Tetrastigma bracteolatum* crude methanol extract on the oral glucose tolerance test (hypoglycemic activity) in mice.

Group	n	Blood glucose level (mean ± SEM) (mmoL/dL)					% Inhibition	
		Fasting	30 min	60 min	120 min	180 min	120 min	180 min
Control (normal saline)	5	4.33 ± 0.82	20.97 ± 1.88	14.57 ± 5.14	15.73 ± 0.03	14.40 ± 0.35	-	-
Glibenclamide (std.) (5 mg/kg)	5	4.13 ± 0.20	12.13 ± 3.03	6.57 ± 0.69*	5.03 ± 1.76**	3.97 ± 0.09***	68.02%	72.4%
MESF (200 mg/kg)	5	4.87 ± 0.27	10.53 ± 1.16**	6.40 ± 1.39*	5.40 ± 0.56***	4.07 ± 0.69***	65.67%	71.74%
MESF (400 mg/kg)	5	5.70 ± 0.57	12.6 ± 2.14*	5.67 ± 0.93*	5.87 ± 0.23***	4.33 ± 0.62***	62.68%	69.93%

Here, *P < 0.05, **P < 0.01 and ***P < 0.001 when compared with the control group. SEM, standard error of mean; MESF, methanol soluble fraction, n: Sample size.

### Effect of METB on CNS-stimulating activity in the thiopental sodium-induced sleeping time bioassay

In the Thiopental sodium–induced sleeping time test, the onset of sleeping was found to be significantly delayed in the treated groups compared to the control: 54.67 ± 15.2 min vs. 10.00 ± 0.0 (P < 0.05) at a METB dose of 200 mg/kg and 37.67 ± 10.11 min vs. 10.00 ± 0.0 at a METB dose of 400 mg/kg (Table 4). The total sleeping time in the treated groups was 137.3 ± 34.7 min (200 mg/kg) and 138.4 ± 14.5 min (400 mg/kg), which were insignificantly lower than that in the untreated group (173.3 ± 3.5 min) (Table 4).

**TABLE 4:** Effects of *Tetrastigma bracteolatum* crude methanol extract on Thiopental sodium-induced sleep in mice (CNS stimulant activity).

Groups	n	Time of onset of sleep (minutes)	Total sleeping time (minutes)
Control (normal saline)	5	10 ± 0.0	173.33 ± 3.48
MESF (200 mg/kg)	5	54.67 ± 15.19***	137.33 ± 34.68*
MESF (400 mg/kg)	5	37.67 ± 10.11**	138.35 ± 14.52**

Here, *P < 0.05, **P < 0.01 and ***P < 0.001 when compared with the control group. SEM, standard error of mean; MESF, methanol soluble fraction, n: Sample size.

### Central analgesic activity of METB

In the case of the central analgesic activity assay by the tail-flick method, the tail flick response time to heat was measured at 0, 30, 60 and 90 min. The latency periods of the METB 200 mg/kg and 400 mg/kg treated mouse groups were found to be significantly prolonged compared to the control. The latency period of 200 mg/kg METB-treated mice was found to be 3.58 ± 0.27 (P < 0.01) at 30 min, 5.50 ± 0.33 (P < 0.001) at 60 min, and 8.71 ± 0.37 (P < 0.001) at 90 min. The percent (%) elongation of response time in mouse groups treated with METB 200 mg/kg, 400 mg/kg, and morphine (2 mg/kg) at 90 min was found to be 275.43%, 298.28% and 334.05%, respectively (Table 5).

**TABLE 5:** Effects of *Tetrastigma bracteolatum* crude methanol extract on tail flicking time of mice (Central analgesic activity).

Group	n	Latency period				% Elongation		
		0 min	30 min	60 min	90 min	30 min	60 min	90 min
Control (normal saline)	5	2.38 ± 0.11	1.97 ± 0.08	2.32 ± 0.14	2.32 ± 0.14	-	-	-
Morphine (2 mg/kg)	5	2.34 ± 0.01	7.74 ± 0.29***	10.07 ± 0.33***	10.07 ± 0.33***	292.89	334.05	334.05
MESF (200 mg/kg)	5	2.25 ± 0.12	3.58 ± 0.27**	5.50 ± 0.33***	8.71 ± 0.37***	81.73	137.07	275.43
MESF (400 mg/kg)	5	2.26 ± 0.18	3.00 ± 0.32*	6.55 ± 0.01***	9.24 ± 0.10***	52.28	182.33	298.28

Here, *P < 0.05, **P < 0.01 and ***P < 0.001 when compared with the control group. SEM, standard error of mean; MESF, methanol soluble fraction; n, Sample size.

### Peripheral analgesic activity of METB

In the acetic acid-induced writhing assay, the number of writhing in mice treated with METB at doses of 200 mg/kg (8.67 ± 0.67, P < 0.001), 400 mg/kg (6.67 ± 0.33, P < 0.001), and the standard analgesic drug diclofenac sodium at a dose of 50 mg/kg (4.67 ± 0.33, P < 0.001) was significantly reduced compared to the untreated mouse group. The highest inhibition of writhing was recorded in mice treated with METB 400 mg/kg (67.74%), which was comparable with that of the standard analgesic drug diclofenac sodium (77.42%) (Table 6).

**TABLE 6:** Effects of *Tetrastigma bracteolatum* crude methanol extract on acetic acid-induced writhing in mice (Peripheral analgesic activity).

Groups	n	Number of writhing by 10 min	Inhibition of writhing (%)
Control (normal saline)	5	20.67 ± 3.48	-
Diclofenac sodium (Std.) (50 mg/kg)	5	4.67 ± 0.33***	77.42
MESF (200 mg/kg)	5	8.67 ± 0.67***	58.06
MESF (400 mg/kg)	5	6.67 ± 0.33***	67.74

Here, *P < 0.05, **P < 0.01 and ***P < 0.001 when compared with the control group. SEM: standard error means, MESF, methanol soluble fraction; n, Sample size.

### Molecular docking analysis

In the field of drug development research, molecular docking prediction has become an extremely prevalent and necessary application. It is able to identify both the conformation of the ligand-binding site on the receptors as well as the method through which it occurs. In the present experiment, molecular docking was carried out with the help of AutoDock Vina software (Pawar and Rohane, 2021; Kumer et al., 2021). Any drug molecule having −6.00 kcal/mol is considered a potential drug candidate (Kumer et al., 2022a; Akash et al., 2022). In our current *in silico* investigation, we performed molecular docking against human CYP3A4 bound to metformin (PDB ID 5G5J), human dipeptidyl peptidase-IV (PDB ID: 4A5S), cyclo-oxygenase-1 (PDB ID 6Y3C), cyclo-oxygenase-2 (PDB ID 1PXX), antioxidant (PDB ID 6NGJ), serotonin receptor 5HT3 (PDB ID 6HIQ), and adenosine caffeine binding receptor (PDB ID 5MZP) (Table 7).

**TABLE 7:** Summary of molecular docking results.

S/N	Human CYP3A4 bound to metformin (PDB ID 5G5J)	Human dipeptidyl peptidase-IV (PDB ID: 4A5S)	Cyclo-oxygenase-1 (PDB ID 6Y3C)	Cyclo-oxygenase-2 (PDB ID 1PXX)	Antioxidant (PDB ID 6NGJ)	Serotonin receptor 5HT3 (PDB ID 6HIQ)	Adenosine caffeine binding receptor (PDB ID 5MZP)
	Binding affinity (kcal/mol)	Binding affinity (kcal/mol)	Binding affinity (kcal/mol)	Binding affinity (kcal/mol)	Binding affinity (kcal/mol)	Binding affinity (kcal/mol)	Binding affinity (kcal/mol)
1	−8.4	−7.4	−8.2	−7.8	−8.9	−7.4	−8.2
2	−5.5	−4.9	−5.5	−5.3	−5.5	−4.8	−5.0
3	−5.9	−5.2	−6.0	−5.5	−7.1	−5.2	−5.6
4	−5.0	−5.1	−5.2	−5.8	−5.6	−4.8	−4.9
5	−7.1	−6.5	−7.2	−6.5	−7.4	−7.8	−6.3
6	−7.5	−7.7	−7.7	−7.5	−8.7	−6.7	−6.5
7	−6.4	−6.0	−6.2	−6.7	−7.5	−6.2	−7.2
8	−8.2	−7.5	−7.7	−8.7	−7.9	−8.0	−7.9
9	−7.5	−6.5	−6.2	−6.3	−7.7	−7.6	−7.6
10	−8.2	−7.4	−7.9	−8.6	−6.9	−8.1	−8.1
11	−7.6	−6.4	−6.5	−7.1	−7.7	−7.5	−7.6
12	−9.0	−10.1	−9.0	10.4	−10.5	−11.7	−9.6
13	−5.6	−4.9	−5.1	−4.8	−6.0	−5.8	−5.8
14	−5.7	−5.2	−4.7	5.1	−6.1	−6.7	−6.8

The maximum binding energies reported for human CYP3A4 bound to metformin (PDB ID 5G5J) are approximately −9.0 kcal/mol and −8.4 kcal/mol, for human dipeptidyl peptidase-IV (−10.1 kcal/mol and −7.1 kcal/mol), for cyclooxygenase-1 (−9.0 kcal/mol and −8.2 kcal/mol), and for cyclooxygenase-2 (−8.7 kcal/mol and −10.4 kcal/mol). Second, the maximum binding affinity is found to be −10.5 kcal/mol and −8.9 kcal/mol against the antioxidant, −8.1 kcal/mol and −11.6 kcal/mol against the serotonin receptor, and −8.2 kcal/mol and −9.6 kcal/mol against the adenosine caffeine binding receptor. In most cases, ligand 01 (6-chloro-4-methyl-2-oxo-2H-chromen-7-yl n-dimethyl carbamate) and ligand 12 (Icosahydro-1,4:5,12:6,11:7,10-tetramethanodibenzo [b,h]biphenylene) are clearly visible.

### Binding interaction and active amino acid analysis

**FIGURE 6:**
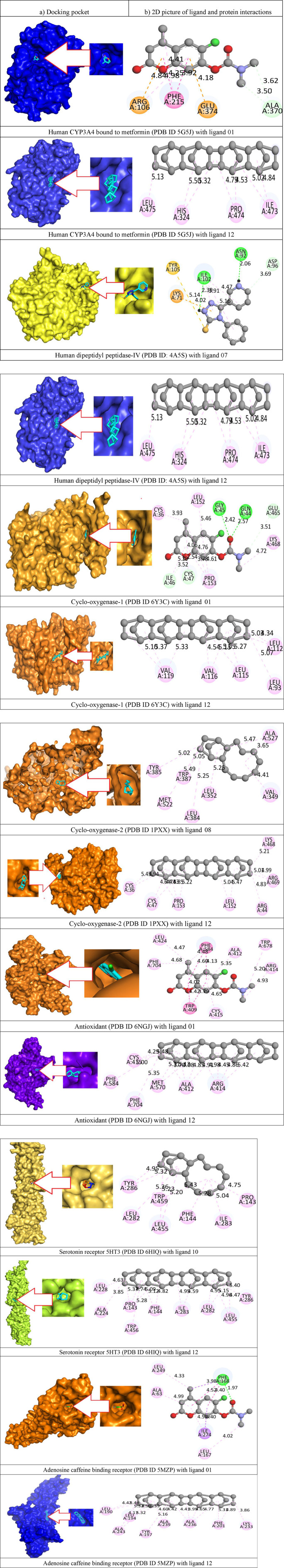
Docking interactions between the proposed compound and 2D picture of active sites. **(a)** Docking pocket. **(b)** 2D picture of ligand and protein interaction.

The interaction between proteins and ligands plays a key part in the process of developing a novel medicine because it provides essential information on the binding or bonding of therapeutics with pathogen-specific proteins. On the other hand, it is an extremely important part of the therapeutic objective. It is now considered to be one of the most difficult areas of drug development owing to the special structural properties of protein interactions with ligands (Kawsar et al., 2022). Biovia Discovery Studio 2021 has developed a tool to visualize protein‒ligand interactions. In this section, of the manuscript, it is shown how a particular protein binds with the molecules, as well as how much of an active site is present following molecular docking experiments. Two figures have been made for the purpose of graphical illustration (Figure 6). The active amino acid residues are found ARH A:106, PHE A:115, GLU A:374, ALA A:370, LEU A:475, PRO A:474, ILE A:473, HIS: 324, TYR A:105, ILE A:92, ILE A:102, LYS A: 71, ASN A: 106, ASP A:96 inn most of the cases. Details active amino acid residues are given in Figure 6.

### ADME/T properties

Lipinski set 5 parameters to be calculated for drug likeliness. If a compound molecular weight <500 amu, hydrogen bond donor site <5, hydrogen bond acceptor site <10, and lipophilicity value LogP ≤5, then the compound would be considered orally bioavailable (Kumer et al., 2022a). In our present studies, all the molecules were satisfied by Lipinski, excluding ligand 14 (phytyl palmitate), since the obtained parameters were within the standard ranges (Table 8).

**TABLE 8:** ADME/T Properties of phytocompounds of *Tetrastigma bracteolatum*.

Lipinski’s rule						ADMET						
S/N	Molecular weight (g/mol)	H-bond acceptor	H-bond donor	LogP	Lipinski’s rule	G.I absorption rate (%)	BBB	CYP450 1A2 inhibitor	CYP450 2C9 substrate	Total clearance (mL/min/kg)	AMES toxicity	Hepatotoxicity
01	281.6	4	0	2.18	Yes	94.69	Yes	Yes	No	0.194	Yes	Yes
02	142.2	2	1	1.04	Yes	98.56	No	No	No	0.583	No	No
03	120.1	1	0	2.47	Yes	99.87	Yes	Yes	No	0.255	No	No
04	110.1	2	2	0.79	Yes	77.19	No	No	No	0.549	No	No
05	342.3	5	0	1.52	Yes	96.18	No	Yes	No	0.863	No	No
06	254.3	2	1	1.51	Yes	95.81	Yes	Yes	No	0.090	Yes	No
07	194.2	2	1	2.65	Yes	95.29	Yes	Yes	No	0.967	No	No
08	283.3	3	1	2.44	Yes	97.01	Yes	Yes	No	0.268	No	Yes
09	134.2	0	0	3.27	Yes	98.22	No	Yes	No	0.173	No	No
10	202.3	0	0	4.33	Yes	98.14	Yes	Yes	No	1.034	No	No
11	282.4	3	1	3.70	Yes	96.02	Yes	Yes	No	1.012	No	No
12	320.5	0	0	7.85	Yes	100	Yes	Yes	No	0.145	No	No
13	226.4	0	0	6.44	Yes	93.56	Yes	Yes	No	1.651	No	No
14	534.9	2	0	8.20	No	88.60	Yes	No	No	1.773	No	No

Second, the ADMET parameters are determined to eliminate unnecessary dangers during preclinical studies. “We know that ADMET assessment is crucial to identify whether or not a molecule is eligible to continue to the clinical stage since drug discovery failures might emerge from effectiveness or safety concerns for scientists. ADMET is also determined by pkCSM online tools, where all the molecules have a high GI absorption rate since the value of GI absorption is greater than 70% (Kumer et al., 2022b). In addition, most drugs easily cross the blood brain barrier and may actively inhibit CYP450 1A2 inhibitors. The total clearance ranges were 0.090 mL/min/k- 1.034 mL/min/kg. Finally, only two drugs may produce AMES toxicity, carcinogenic effects, and hepatotoxic effects. Therefore, overall pharmacokinetic parameters are accepted and might have suggested them as potential drug candidate.

## Discussion

The highest phenolic contents were observed in the CSF and DCMSF fractionates of T. bracteolatum. This high phenolic content could be the cause for the antioxidant activities of the extracts (Grzegorczyk-Karolak et al., 2015; Pietta, 2000). In our study, it was found that the CSF (IC_50_: 27.74 ± 0.55 μg/mL) and AQSF (IC_50_: 7.48 ± 0.05 μg/mL) exhibited potential antioxidant activity to scavenge DPPH free radicals. In a previous study, Islam et al. (2023) also reported potential antioxidant activity of ethanol crude extract of *T. bracteolatum* (IC_50_: 56 μg/mL). Therefore, the antioxidant activity of *T. bracteolatum* and/or its fractions is supported by previous findings (Islam et al., 2023). However, likewise the findings of Islam et al., the methanol crude extract in our study also showed lower potential for antioxidant activity (IC_50_: 170.13 ± 2.40 μg/mL) and potential antioxidant activities were shown in fractions. Interestingly, the AQSF (aqueous soluble fraction of extract) (IC_50_: 7.48 ± 0.05) was found to be as a stronger antioxidant than that of BHT (a standard antioxidant used in this experiment) (IC_50_: 21.68 ± 0.41). The highest cytotoxic activity in the brine shrimp bioassay was observed by CSF (LC50: 1.41), whereas the LC50 of the standard anticancer drug vincristine sulfate was 0.45 (Table 1). This finding indicates that CSF possesses a potential bioactive compound(s) responsible for cytotoxic activity. The potential antioxidant activity and the presence of a high number of phenolic compounds may be the possible reason behind the strong cytotoxic activities of the CSF fraction of T. bracteolatum. Membrane stabilizing activity is likely to hamper the inflammatory response and thus is expected to exhibit anti-inflammatory activity. Even though, in both cases, the results were lower but promising when compared to the standards, the different fractionates were lower than the standard streptokinase. One of the main factors responsible for the inflammatory response in the body is the release of cytokines, which increase the migration of WBCs and further contribute to the inflammatory cascade. The lysosomal membrane stores cytokines and other inflammatory mediators and can be compared to the membrane of an erythrocyte. Stabilization of this membrane could be an important factor in limiting the inflammatory response by preventing the release of lysosomal constituents of activated neutrophils, such as bactericidal enzymes and proteases, which cause further tissue inflammation and damage upon extracellular release (Babu et al., 2011). The CSF and MESF fractionates exhibited promising thrombolytic activity, which may be valuable for the discovery of new drugs in the management of cardiovascular diseases. In the case of both the hypotonic solution and heat-induced hemolysis, the CSF showed potential membrane stabilizing activities (Figure 4). D*ia*rrhea refers to the excess passage of watery stools resulting from a decrease in consistency or an increased frequency of movement of the bowl. Castor oil initiates diarrhea by stimulating intestinal motility and secretory processes (Zhou et al., 2024), and induction of diarrhea by castor oil is a common method that is being followed in this current study. A study conducted by Islam et al (2023) on the antidiarrheal effect of ethanol crude extract 250 mg/kg and 500 mg/kg doses on experimental mice resulted the inhibition of 28.8% and 51.5% defecation in castor oil induced diarrhea (Islam et al, 2023). Likewise the previous study, our investigation resulted more potential antidiarrheal activities of methanol extract. The methanol extract at doses of 200 and 400 mg/kg showed 80.78% (P < 0.001) and 87.18% (P < 0.001) of defecation in diarrhea. Loperamide (3 mg/kg) was used as the positive control which inhibited diarrhea by 82.05%. This dose-dependent inhibition of diarrheal stool indicates the presence of bioactive compounds capable of antidiarrheal activity. Diabetes is a disease condition caused when the body’s ability to process blood glucose is impaired due to either a lack of secretion or the action of insulin (Chen et al., 2019; Sarker et al., 2015). Antihyperglycemic agents decrease blood glucose levels either by increasing glucose uptake or by potentiating insulin secretion from the pancreas (Rouhi et al., 2017; Shah et al., 2016). The crude extract of the plant showed significant (P < 0.001) time-dependent hypoglycemic activity at both doses (200 and 400 mg/kg). The extract at two dif-ferent doses reduced the blood glucose level by 71.74% and 69.93% when compared to the control, and the standard glibenclamide showed 72.4% inhibition. This result indicates that the crude extract of the plant *T. bracteolatum* may be a potential source of antihyperglycemic agents. Our antidiabetic result is also supported by the findings reported by Islam et al. (2023). They found that the ethanol extract of *T. bracteolatum* at the dose of 250 and 500 mg/kg reduced plasma glucose level 90 min after the administration of glucose load in a dose dependent manner (Islam et al., 2023). The CNS-stimulating activity of the crude methanol extract of the plant (METB) was also promising. The extract at a dose of 200 mg/kg potently delayed the sleeping time of the experimental mice (54.67 ± 15.2 min vs. 10.00 ± 0.0) (Table 4). In the current study, the crude extract of the plant showed significant (P < 0.001) dose-dependent inhibition of acetic acid-induced writhing *in-vivo*. However, the results are lower compared to the NSAID diclofenac sodium. The writhing response initiated by acetic acid may cause the release of inflammatory mediators such as prostaglandins and prostacyclins, which are responsible for the sensitization of nerve fibers, resulting in pain sensations (Nesa et al., 2018; Eghianruwa et al., 2020). Inhibition of the writhing response is an indication that the plant extract of *T. bracteolatum* may produce nonnarcotic analgesic activity due to the inhibition of prostaglandin synthesis by blocking lipoxygenase and cyclooxygenase activities. The crude extract of *T. bracteolatum* showed dose-dependent inhibition of analgesic activity both centrally and peripherally. The ‘tail flick’ method for the central and the ‘writhing technique’ for the peripheral analgesic activity are very useful techniques for the evaluation of analgesic activity of any plant extract (Fan et al., 2014). The central analgesic action is mediated via inhibition of central pain receptors (Ohashi and Kohno, 2020). It is now evident that μ, κ3, d and d2 are the opioid receptor subtypes primarily responsible for the supraspinal-mediated analgesic action of opiates, and spinal analgesia appears to be mediated through μ2, d2 and κ1 receptors (Reeves et al., 2022). A previous study reported the similar findings on the analgesic activity of the ethanol extract of *T. bracteolatum* in acetic acid-induced writhing method using mice which resulted the inhibition of writhing by 28.8% and 51.5% at the doses of 250 and 500 mg/kg, respectively (Islam et al., 2023). Therefore, our reported analgesic activity is supported by the previous similar study. Opiates such as morphine and its derivatives (plant origin) exert their analgesic activity by interacting with various receptors both at spinal and supraspinal sites. Morphine at a dose of 2 mg/kg.b.wt. caused a significant analgesic effect in the tail flick method, and the effect became more prominent as time increased. Similar effects were recorded in the case of the methanolic extracts of the plant. On the other hand, the peripheral analgesic effect is generally mediated through inhibition of cyclooxygenase and/or lipoxygenase and other inflammatory mediators or inhibition of pain responses peripherally mediated by nociceptors. Therefore, it is possible that the methanolic extract of this plant may show an analgesic effect through these mechanisms. Both the central and peripheral analgesic effects increased with increasing dose and duration of action in the mouse model. Although the chemical(s) responsible for this analgesic activity and the mechanism of action (interactions with receptors or other mediators) are still unknown, the promising result demands further chemical investigation to determine the active principle(s) responsible for the analgesic activity. In addition, different types of computational studies are also performed in this current investigation, including molecular docking and ADMET studies. In molecular docking studies, most of the compounds have reported greater binding energy to be potential drug candidates. The ADMET properties are accepted by all molecules and are reported to be free from any type of carcinogenic effects or hepatotoxic effects. Finally, different active amino acids are seen during the formation of the drug-protein complex, including ARH A:106, PHE A:115, GLU A:374, ALA A:370, LEU A:475, PRO A:474, ILE A:473, HIS: 324, TYR A:105, ILE A:92, ILE A:102, LYS A: 71, ASN A: 106, and ASP A:96 in most cases. Details of the active amino acid residues are given in Figure 4.

The experimental evidences of this study demonstrated that the methanol extract of *T. bracteolatum* possess potential antidiabetic, anti-oxidant, antidiarrheal, analgesic, and CNS stimulating activities. In silico molecular docking explored the mechanism of actions for those effects and supported the potentiality of the extract to exhibit those effects. In most the pharmacological activities of the extract was exhibited due to the presence of the mainly 2 phytocompounds: namely ligand 01 (6-chloro-4-methyl-2-oxo-2H-chromen-7-yl n-dimethyl carbamate) and ligand 12 (Icosahydro-1,4:5,12:6,11:7,10-tetramethanodibenzo [b,h]biphenylene). The compound 12 is the most prominent bioactive compound which has showed the highest binding score with the targeted receptors (binding scrore for metformin receptor was 9.0 and DPP-4 inhibitors receptor 10.1). Thus, the antidiabetic activity of methanol extract of *T*. *bracteolatum* mechanism is similar with the molecular mechanism of metformin and DPP-4 inhibitors. The other mechanisms of the extract for antioxidant, analgesic, CNS stimulation activities are shown through cyclooxygenase-1 and cyclooxygenase-2 receptors, Serotonin receptor (5-HT3), and caffeine receptors due to the presence of compound 1 and compound 12. Thus, the findings of the our study would be a prospects for the discovery and development of antidiabetic, analgesic, antidiarrheal, and CNS stimulating novel drugs or the standardized extract of the plant can be used for the complementary and/or alternative medicine for the treatment of those diseases. However, further extensive preclinical, clinical and toxicological investigations are required for its medication use.

## Conclusion

This study aimed to scientifically validate the traditional use of *Tetrastigma bracteolatum* through a range of *in vitro*, *in vivo*, and *in silico* assays. Among the tested fractions, the chloroform-soluble fraction (CSF) exhibited the most potent cytotoxic, membrane-stabilizing, and thrombolytic activities. The methanolic crude extract also demonstrated significant antidiabetic, antidiarrheal, CNS-stimulating, and analgesic properties in animal models.

To the best of our knowledge, this is the first comprehensive pharmacological assessment of *T. bracteolatum*, supporting its ethnomedicinal use - particularly for pain management by confirming the presence of phytoconstituents with analgesic and anti-inflammatory potential. These findings highlight the plant’s promise as a source of novel therapeutic agents.

Further research is warranted to isolate and characterize the active compounds responsible for these activities and to perform detailed mechanistic and safety evaluations. Such investigations may pave the way for the development of new, plant-based drug candidates derived from *T. bracteolatum*.

## Data Availability

The original contributions presented in the study are included in the article/supplementary material, further inquiries can be directed to the corresponding authors.
